# BAK-preserved hyaluronate and corneal nerves

**DOI:** 10.22336/rjo.2026.53

**Published:** 2026

**Authors:** Radu Bucsan, Ruxandra Pîrvulescu, Mădălina Totir, Ruxandra Coroleucă, Lavinia-Maria Olteanu, Alina Popa-Cherecheanu, Andreea Popa-Cherecheanu, Dan George Deleanu

**Affiliations:** 1 Department of Ophthalmology, “Carol Davila” University of Medicine and Pharmacy, Bucharest, Romania; 2 Department of Ophthalmology, Emergency University Hospital Bucharest, Bucharest, Romania

**Keywords:** dry eye disease, benzalkonium chloride, hyaluronic acid, in vivo confocal microscopy, corneal subbasal nerve plexus, BAK = benzalkonium chloride, BAK HA = benzalkonium chloride-preserved hyaluronic acid, CFracDim = corneal nerve fractal dimension, CI = confidence interval, CNBD = corneal nerve branch density, CNFA = corneal nerve fiber area, CNFD = corneal nerve fiber density, CNFL = corneal nerve fiber length, CNFW = corneal nerve fiber width, CTBD = corneal total branch density, DED = dry eye disease, FCS = fluorescein corneal staining, HA = hyaluronic acid, IVCM = in vivo confocal microscopy, LCS = lissamine green conjunctival staining, NIBUT = non-invasive tear break-up time, OSDI = Ocular Surface Disease Index, PF HA = preservative-free hyaluronic acid, TBUT = tear break-up time with sodium fluorescein, TFOS DEWS II = Tear Film and Ocular Surface Society Dry Eye Workshop II, TFOS DEWS III = Tear Film and Ocular Surface Society Dry Eye Workshop III

## Abstract

**Objective:**

Benzalkonium chloride (BAK) is the most commonly used preservative in multidose ophthalmic solutions, and experimental studies indicate that it damages corneal nerves in a concentration- and duration-dependent manner. No randomized trial has examined whether BAK-preserved artificial tears alter the corneal subbasal nerve plexus in dry eye disease. This analysis asks whether six months of exposure to BAK at the concentration used in artificial tears is associated with detectable alterations in corneal innervation.

**Methods:**

In this substudy of a six-month, randomized, single-masked trial, imaging was a protocol-defined secondary objective. We compared 0.15% sodium hyaluronate preserved with 0.005% BAK (BAK HA) with the preservative-free formulation of the same product (PF HA), both instilled pro re nata. Thirty-two of the 43 trial completers underwent in vivo confocal microscopy of the central cornea at baseline and at six months, with 19 receiving PF HA and 13 receiving BAK HA. We analyzed seven corneal nerve parameters using mixed-effects models that accounted for both eyes.

**Results:**

Baseline characteristics and all baseline nerve parameters were comparable. Over six months, no parameter showed a statistically significant between-group difference in change. For corneal nerve fiber density, the change in the BAK HA group exceeded that in the PF HA group by 3.52 fibers/mm² (95% confidence interval, −2.32 to 9.37; P = 0.238).

**Discussion:**

Our design removes the active drug that confounds the glaucoma literature, but the estimates are imprecise, and the design cannot establish equivalence.

**Conclusions:**

This substudy detected no between-group difference in corneal subbasal nerve parameters.

## Introduction

Dry eye disease (DED) is a prevalent, multifactorial disorder of the tear film and ocular surface, with an estimated global prevalence of 12% [1-4]. It is characterized by loss of tear film homeostasis and clinically presents with ocular discomfort, burning, dryness, irritation, and variable vision. These symptoms substantially impair visual function, reduce quality of life, and contribute to sleep disturbances and psychological distress [5-10]. Consequently, DED imposes a substantial socioeconomic burden on patients and healthcare systems [11,12].

The pathophysiology of DED involves a complicated interplay among tear film instability, hyperosmolarity, ocular surface inflammation, epithelial damage, and, in some cases, neurosensory dysfunction [13-15]. Osmotic and inflammatory stress often drives these mechanisms, leading to elevated levels of proinflammatory cytokines and apoptotic markers on the ocular surface [16-22]. Because the course is chronic and often progressive, DED requires long-term topical therapy, and artificial tears remain the mainstay of treatment [23]. However, many commercially available formulations contain preservatives, particularly benzalkonium chloride (BAK), which can cause cumulative ocular surface toxicity with prolonged use [23]. Experimental and observational studies have consistently reported BAK-related adverse effects, including conjunctival inflammation, goblet cell loss, and epithelial barrier disruption [24-29]. Few randomized controlled trials compare preservative-free and BAK-preserved artificial tears having similar concentrations, molecular weight, and excipients, and these differences confound the attribution of clinical outcomes to the preservative.

In vivo confocal microscopy (IVCM) allows real-time, high-resolution imaging of the ocular surface at the cellular level [30]. It allows detailed assessment of epithelial cells, keratocytes, the subbasal nerve plexus, dendritic cells, and endothelial cells [31,32]. In DED, IVCM has revealed changes in keratocyte morphology, reduced subbasal nerve density, and increased dendritic cell presence, indicating the disease’s inflammatory and metabolic disturbances [33-35]. These measurements provide objective biomarkers of disease severity and evolution and can be used to monitor treatment response [14,15,36,37].

Researchers have examined corneal nerves in particular with respect to preservative exposure. In animal models, topical BAK reduces corneal nerve density in a manner that depends on both the concentration applied and the duration of exposure. The reduction extends from the superficial intraepithelial terminals to the subbasal plexus and, at the highest concentrations, to the stromal nerves [38,39]. A single instillation of BAK on a debrided murine cornea reduces stromal nerve volume and mechanosensation [40], and continuous exposure at clinically used concentrations impairs the activity of corneal cold-sensitive neurons in the rat without subsequent functional recovery [41]. Sodium hyaluronate, in contrast, attenuates BAK-associated epithelial and neuronal toxicity and accelerates recovery of corneal mechanical sensitivity in mice [42].

In humans, most available evidence comes from patients receiving chronic topical therapy for glaucoma. IVCM studies of these patients have reported alterations in subbasal nerve morphology, including fewer main nerve trunks, increased tortuosity and beading, altered traced nerve length, and increased dendritic cell density [43-46]. We cannot separate these associations from the effects of the active drug, the number of concomitant medications, the number of daily instillations, disease severity, and coexisting ocular surface disease. In healthy volunteers, twelve weeks of exposure to 0.01% BAK alone produced a reversible increase in corneal Langerhans cell density, although corneal nerves were not quantified [47]. To our knowledge, no randomized trial of BAK-preserved artificial tears has reported corneal nerve outcomes. However, randomized comparisons of preserved and preservative-free formulations have assessed other ocular surface endpoints.

We therefore analyzed corneal nerve outcomes from a randomized, single-masked, six-month clinical trial comparing preservative-free hyaluronic acid (PF HA) with BAK-preserved hyaluronic acid (BAK HA) in patients with DED [48]. IVCM was listed among the secondary objectives in the protocol approved by the ethics committee before recruitment began, and we report the imaging results here as a separate substudy. Both formulations contained the same concentration and molecular weight of sodium hyaluronate. The same manufacturer produced both formulations, so the two arms received closely matched formulations that differed primarily in preservative content. The trial’s primary outcome, the change in the Ocular Surface Disease Index (OSDI©) score, and the clinical signs of DED are reported separately [48]. This analysis asks whether six months of exposure to BAK at the concentration used in artificial tears is associated with detectable alterations in corneal innervation.

## Materials and methods

### Study design and participants

This analysis reports a secondary outcome of a six-month, randomized, single-masked clinical trial conducted in accordance with the Declaration of Helsinki and the European Union’s Good Clinical Practice guidelines. The Ethics Committee of the Emergency University Hospital Bucharest approved the protocol (ID: 37003, approval date June 2022), and all participants provided written informed consent at the screening visit after we explained the study’s nature and possible consequences. We reported the trial design, eligibility criteria, and clinical outcomes separately [48]. IVCM was listed as a secondary objective in the approved protocol before recruitment began.

Participants were 18 years of age or older. They had a documented history of DED lasting more than three months, as defined by the diagnostic criteria in the TFOS DEWS II Diagnostic Methodology report. They also had a tear break-up time of 10 seconds or less, a Schirmer I test result of 15 mm or less, and at least one DED symptom, such as burning or stinging, foreign body sensation, blurred vision, a sticky feeling, photosensitivity, itching, or pain [15]. Ophthalmic findings were otherwise normal, and no topical lubricant had been administered in the 24 hours preceding the screening visit. Patients were excluded if they had clinically significant slit-lamp findings at screening, abnormal lid anatomy or blinking function, DED secondary to scarring, contact lens wear within the preceding three months or anticipated during the study, a history of glaucoma, corticosteroid treatment within the preceding four weeks, treatment with parasympathomimetic or antipsychotic drugs, topical treatment with any ophthalmic drug other than lubricants within the preceding four weeks, ocular surgery within the preceding three months or planned within the following six months, punctum plugs, Stevens-Johnson syndrome, active ocular allergy or ocular allergy expected to be active during the study period, participation in another clinical trial within the preceding four weeks, or pregnancy, planned pregnancy within 12 months, or lactation.

Forty-seven patients were randomized in the parent trial, 24 to PF HA and 23 to BAK HA, and 43 completed the six-month visit [48]. Two participants in each group did not attend the final visit. IVCM was offered to all participants at the baseline visit. Thirty-two participants were examined at both the baseline and six-month visits and are included here: 19 in the PF HA group and 13 in the BAK HA group. Eleven completers, 3 in the PF HA group and 8 in the BAK HA group, declined the examination or could not tolerate it at the baseline visit, before treatment allocation; therefore, these decisions could not have been influenced by knowledge of the assigned treatment. All 11 were women. No participant was excluded because of image quality, although we excluded two eyes from the primary analysis for that reason, as described under Statistical analysis. **Fig. 1** shows the participant flow. We treated and imaged both eyes of each participant.

**Fig. 1:**
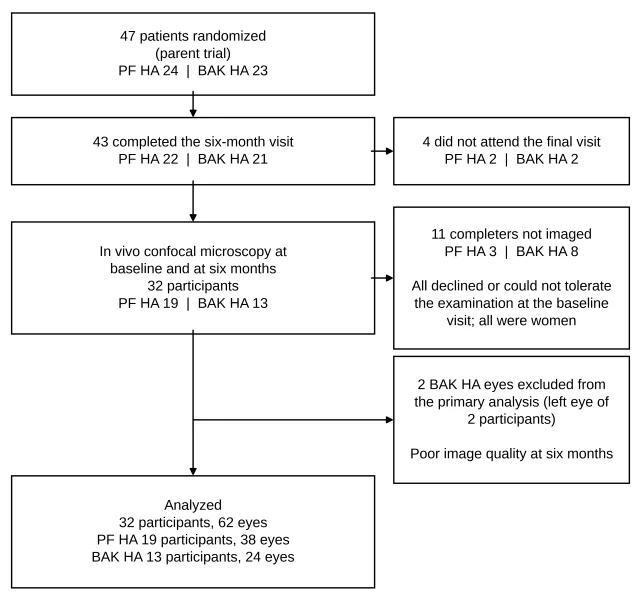
Flow of participants from the parent trial’s randomized cohort into the in vivo confocal microscopy substudy. PF HA, preservative-free hyaluronic acid; BAK HA, benzalkonium chloride-preserved hyaluronic acid

We randomized using software in a 1:1 ratio without stratification. The clinical examiner, the IVCM operator, and the person who selected the frames for analysis were masked to treatment allocation.

### Interventions

Two artificial tears, available in both single-dose, preservative-free and multidose, BAK-preserved presentations, were compared to ensure excipient differences would not confound the comparison. The same company manufactured both products, and they were identical in composition except for the preservative. Lubristil (SIFI S.p.A., Italy), referred to here as BAK HA, contains sodium hyaluronate 0.15%, sodium chloride, disodium phosphate dodecahydrate, sodium phosphate monobasic monohydrate, benzalkonium chloride 0.005%, and purified water. Lubristil single-dose, preservative-free (SIFI S.p.A., Italy), referred to here as PF HA, contains sodium hyaluronate 0.15%, sodium chloride, disodium phosphate, monobasic sodium phosphate, and distilled water. The mean molecular weight of hyaluronic acid in both formulations is 1.5 to 3.0 MDa. Participants were randomized to one of the two formulations and instilled it pro re nata in both eyes throughout the six-month study period. Participants recorded each instillation in a personal diary, and no maximum daily number was imposed [48]. Participants were instructed not to administer the study treatment within two hours preceding a study visit and to report adverse events at any time. The study comprised four visits: baseline, one month ± 1 week, three months ± 1 week, and six months ± 1 week.

### In vivo confocal microscopy

We acquired corneal images using a Heidelberg Retina Tomograph III with the Rostock Cornea Module. This 670 nm diode laser system provides optical sections approximately 4 micrometers thick within a 400 by 400 micrometer field of view. We performed IVCM at baseline and at the six-month visits. We used topical anesthesia (oxybuprocaine hydrochloride 4 mg/mL, Rompharm) and a coupling gel (Eye Gel Visc Ophtal, Dr. Robert Winzer Pharma GmbH). The examined region was a central zone approximately 2 mm in diameter, centered on the operationally localized corneal apex, and included only the subbasal nerve plexus.

We recorded three image sequences per eye at each visit. From these sequences, we retained one frame per eye for analysis. We selected the retained frame based solely on technical quality, without preferentially selecting fields with more numerous or more visible nerve fibers. The person who selected the frame was masked to treatment allocation and visit. Therefore, each eye and visit contributed a single 400 by 400 micrometer frame, corresponding to a sampled area of 0.16 mm². No universally applicable number of fields has been established for corneal nerve morphometry, and the required number depended on the parameter and the sampling protocol [49,50]; we addressed the consequences of sampling a single frame in the Discussion.

We quantified the nerve parameters using ACCMetrics (version 2, University of Manchester, Manchester, UK), which automatically segments the subbasal nerves using the multi-scale morphometric approach described by Dabbah and colleagues [51]. We used the software-generated segmentation without manual correction. One observer, blinded to treatment allocation and visit, performed the analysis.

Seven corneal nerve parameters were measured for each eye: corneal nerve fiber density (CNFD), corneal nerve branch density (CNBD), corneal nerve fiber length (CNFL), corneal total branch density (CTBD), corneal nerve fiber area (CNFA), corneal nerve fiber width (CNFW), and corneal nerve fractal dimension (CFracDim). CNFD, CNBD, and CTBD are counts of main fibers, primary branches, and all branch points, respectively, each normalized to the imaged area; CNFL is the total length of all traced fibers, normalized to the imaged area; CNFA is the total nerve fiber area, normalized to the imaged area; CNFW is the mean fiber width, also normalized to the imaged area; CFracDim is a dimensionless measure of the plexus’s branching complexity.

### Statistical analysis

We summarized continuous baseline characteristics as mean ± standard deviation and compared them between the two treatment groups using independent-samples t-tests. Sex was summarized as counts and percentages and compared using Fisher’s exact test.

We compared baseline corneal nerve parameters between groups using mixed-effects models with a random intercept for participants to account for both eyes. We analyzed the six-month change in each corneal nerve parameter using longitudinal mixed-effects models with fixed effects for treatment group, visit, and their interaction, and random intercepts for participants and eyes nested within participants to account for bilateral and repeated measurements. The between-group difference reported in **Table 1** is the change from baseline to six months in the BAK HA group minus the corresponding change in the PF HA group. Hence, a positive value indicates a greater increase in the BAK HA group. Missing outcomes were not imputed. The primary analysis included all 38 eyes in the PF HA group and 24 of the 26 eyes in the BAK HA group. We excluded the left eye of two BAK HA participants because the six-month images were of poor quality (**Table 2**). For the within-group changes in **Table 2**, we calculated 95% confidence intervals from participant-clustered standard errors, with a t-distribution based on the number of participant clusters.

**Table 1:** Between-group differences in the six-month change in in vivo confocal microscopy corneal nerve parameters

IVCM parameter	Between-group difference in six-month change	95% CI	P value
Corneal nerve fiber density (CNFD), fibers/mm²	3.52	−2.32 to 9.37	0.238
Corneal nerve branch density (CNBD), branches/mm²	0.38	−6.67 to 7.43	0.915
Corneal nerve fiber length (CNFL), mm/mm²	1.04	−1.31 to 3.39	0.386
Corneal total branch density (CTBD), branch points/mm²	0.65	−10.63 to 11.94	0.910
Corneal nerve fiber area (CNFA), mm²/mm²	0.0003	−0.0009 to 0.0016	0.612
Corneal nerve fiber width (CNFW), mm/mm²	−0.0007	−0.0031 to 0.0017	0.572
Corneal nerve fractal dimension (CFracDim), dimensionless	0.0182	−0.0187 to 0.0552	0.334

*Differences represent the change from baseline to six months in the BAK HA group minus the corresponding change in the PF HA group. Estimates were derived from longitudinal mixed-effects models with fixed effects for treatment group, visit, and their interaction, and random intercepts for participants and eyes nested within participants to account for bilateral and repeated measurements. Positive values indicate a greater increase in the BAK HA group. Because the parameters are expressed in different units, the magnitudes of the estimates are not comparable across rows. All seven outcomes are exploratory and are reported without adjustment for multiple comparisons. The analysis set is the same as in **Table 2**. CI, confidence interval; IVCM, in vivo confocal microscopy; PF HA, preservative-free hyaluronic acid; BAK HA, benzalkonium chloride-preserved hyaluronic acid.*

**Table 2:** Corneal nerve parameters at baseline and at six months, and the within-group change, by treatment group

IVCM parameter	Group	Baseline	Six months	Change (95% CI)	Eyes analyzed, n
Corneal nerve fiber density (CNFD), fibers/mm²	PF HA	10.36 (10.20)	9.70 (8.92)	−0.66 (−5.11 to 3.79)	38
	BAK HA	10.94 (9.62)	13.80 (10.42)	2.86 (−2.01 to 7.74)	24
Corneal nerve branch density (CNBD), branches/mm²	PF HA	7.07 (10.49)	9.29 (11.45)	2.22 (−1.93 to 6.37)	38
	BAK HA	7.81 (11.10)	10.42 (11.46)	2.60 (−3.07 to 8.28)	24
Corneal nerve fiber length (CNFL), mm/mm²	PF HA	8.58 (3.86)	8.50 (4.11)	−0.09 (−1.76 to 1.58)	38
	BAK HA	8.85 (3.12)	9.80 (4.16)	0.95 (−1.07 to 2.97)	24
Corneal total branch density (CTBD), branch points/mm²	PF HA	20.56 (19.00)	20.56 (16.94)	0.00 (−8.36 to 8.36)	38
	BAK HA	22.52 (15.22)	23.18 (18.29)	0.65 (−9.77 to 11.07)	24
Corneal nerve fiber area (CNFA), mm²/mm²	PF HA	0.0045 (0.0020)	0.0041 (0.0020)	−0.0004 (−0.0012 to 0.0005)	38
	BAK HA	0.0046 (0.0015)	0.0046 (0.0018)	−0.0001 (−0.0012 to 0.0011)	24
Corneal nerve fiber width (CNFW), mm/mm²	PF HA	0.0249 (0.0032)	0.0249 (0.0039)	0.0000 (−0.0021 to 0.0021)	38
	BAK HA	0.0238 (0.0030)	0.0231 (0.0028)	−0.0007 (−0.0027 to 0.0013)	24
Corneal nerve fractal dimension (CFracDim), dimensionless	PF HA	1.427 (0.063)	1.418 (0.068)	−0.009 (−0.039 to 0.021)	38
	BAK HA	1.429 (0.047)	1.438 (0.060)	0.009 (−0.021 to 0.039)	24

Values at baseline and at six months are observed means (standard deviation). The change is the six-month value minus the baseline value, reported as the mean (95% CI). The confidence interval uses participant-clustered standard errors to account for the correlation between the two eyes, with a t-distribution based on the number of participant clusters. The primary analysis excludes the left eye of two BAK HA participants at the six-month visit because of poor image quality. CI, confidence interval; IVCM, in vivo confocal microscopy; PF HA, preservative-free hyaluronic acid; BAK HA, benzalkonium chloride-preserved hyaluronic acid.

We did not adjust the comparison for the number of instillations. We measured that variable after randomization; it may be affected by both treatment and symptoms, and adjusting for it would change the quantity being estimated.

All seven corneal nerve parameters are reported as exploratory outcomes. We considered a two-sided P value below 0.05 statistically significant and did not adjust for multiple comparisons. Therefore, the P values should be interpreted as descriptive rather than confirmatory. All analyses were performed using Stata version 16 (StataCorp LLC, College Station, TX, USA).

## Results

### Participant flow

Of the 47 patients randomized in the parent trial, 24 to PF HA and 23 to BAK HA, 43 completed the six-month visit: 22 in the PF HA group and 21 in the BAK HA group. Two participants in each group did not attend the final visit [48]. Thirty-two of these 43 participants underwent IVCM at both the baseline and the six-month visit. They formed the present analysis set, 19 in the PF HA group and 13 in the BAK HA group (**Fig. 1**). The remaining 11 participants, 3 in the PF HA group and 8 in the BAK HA group, declined the examination or could not tolerate it at the baseline visit, before any study treatment had been instilled. All 11 were women, so the number of male participants was identical in the completer cohort and in the imaging substudy in both arms. Because these decisions were made at the baseline visit, treatment allocation, tolerability, or disease course could not have influenced them. Both eyes of each participant were imaged, yielding 38 eyes in the PF HA group and 26 eyes in the BAK HA group. We excluded two BAK HA eyes (the left eyes of two participants) from the primary analysis because the six-month images were of poor quality. Therefore, the analysis includes 38 eyes in the PF HA group and 24 eyes in the BAK HA group. Participants reported no adverse events during the study period [48].

### Baseline characteristics of the confocal microscopy subgroup

Baseline age, sex distribution, OSDI© score, and ocular surface parameters were comparable between the two treatment groups, and none of the differences were statistically significant (all P values were 0.149 or higher; **Table 3**). The proportion of male participants was higher in the PF HA group (12 of 19 [63%]) than in the BAK HA group (4 of 13 [31%]), but this difference was not statistically significant (P = 0.149).

**Table 3:** Baseline characteristics of participants in the in vivo confocal microscopy substudy, by treatment group

Baseline variable	PF HA, n = 19	BAK HA, n = 13	P value
Age, years	47.58 ± 16.31	46.62 ± 15.08	0.865
Male sex, n (%)	12 (63)	4 (31)	0.149
OSDI©	31.60 ± 12.64	36.77 ± 13.25	0.280
Mean bilateral NIBUT, s	9.47 ± 4.76	7.81 ± 2.49	0.209
Schirmer I, mm	9.63 ± 3.73	7.27 ± 4.92	0.157
TBUT, s	5.68 ± 2.30	6.04 ± 1.86	0.635
FCS, Oxford grade	0.92 ± 0.53	1.15 ± 0.66	0.301
LCS, Oxford grade	0.82 ± 0.80	0.69 ± 0.56	0.612

*Values are presented as mean ± standard deviation unless otherwise indicated. P values were calculated using independent-samples t tests for continuous variables and Fisher’s exact test for sex. PF HA, preservative-free hyaluronic acid; BAK HA, benzalkonium chloride-preserved hyaluronic acid; OSDI, Ocular Surface Disease Index; NIBUT, non-invasive tear break-up time; TBUT, tear break-up time; FCS, fluorescein corneal staining; LCS, lissamine green conjunctival staining.*

### Baseline corneal nerve parameters

Baseline IVCM corneal nerve parameters were comparable between the two treatment groups, and none of the differences reached statistical significance (all P values were 0.231 or higher; **Table 4**). Corneal nerve fiber density was 10.36 ± 10.20 fibers/mm² in the PF HA group and 10.34 ± 9.51 fibers/mm² in the BAK HA group (P = 0.993). Corneal nerve fiber length was 8.58 ± 3.86 mm/mm² and 8.52 ± 3.22 mm/mm², respectively (P = 0.948). The smallest P value was observed for corneal nerve fiber width (P = 0.231).

**Table 4:** Baseline in vivo confocal microscopy corneal nerve parameters, by treatment group

IVCM parameter	PF HA, n = 19 participants (38 eyes)	BAK HA, n = 13 participants (26 eyes)	P value
Corneal nerve fiber density (CNFD), fibers/mm²	10.36 ± 10.20	10.34 ± 9.51	0.993
Corneal nerve branch density (CNBD), branches/mm²	7.07 ± 10.49	7.21 ± 10.85	0.959
Corneal nerve fiber length (CNFL), mm/mm²	8.58 ± 3.86	8.52 ± 3.22	0.948
Corneal total branch density (CTBD), branch points/mm²	20.56 ± 19.00	21.03 ± 15.55	0.927
Corneal nerve fiber area (CNFA), mm²/mm²	0.0045 ± 0.0020	0.0046 ± 0.0015	0.865
Corneal nerve fiber width (CNFW), mm/mm²	0.0249 ± 0.0032	0.0239 ± 0.0029	0.231
Corneal nerve fractal dimension (CFracDim), dimensionless	1.427 ± 0.063	1.424 ± 0.053	0.863

*Values are presented as mean ± standard deviation or median (interquartile range), as appropriate. P values were derived from mixed-effects models with a random intercept for participants to account for both eyes. Baseline values in this table include all 26 BAK HA eyes. In contrast, **Table 2** reports the 24 BAK HA eyes retained in the primary analysis, so the BAK HA baseline means differ slightly between the two tables. IVCM, in vivo confocal microscopy; PF HA, preservative-free hyaluronic acid; BAK HA, benzalkonium chloride-preserved hyaluronic acid.*

### Six-month changes in corneal nerve parameters

Over six months, none of the seven IVCM corneal nerve parameters showed a statistically significant between-group difference in change (all P values were 0.238 or higher; **Table 1**). For corneal nerve fiber density, the change in the BAK HA group exceeded that in the PF HA group by 3.52 fibers/mm² (95% confidence interval, −2.32 to 9.37; P = 0.238). The corresponding estimate for corneal nerve fiber length was 1.04 mm/mm² (95% confidence interval, −1.31 to 3.39; P = 0.386). Because the parameters are expressed in different units, we cannot rank the magnitudes of these estimates against one another.

The point estimates for corneal nerve fiber density, branch density, fiber length, total branch density, fiber area, and fractal dimension were positive, showing a numerically greater increase in the BAK HA group. In contrast, the estimate for corneal nerve fiber width was negative. None of these differences were statistically significant, and every confidence interval included zero, so these estimates should not be interpreted as a treatment effect. We analyzed the seven parameters as exploratory outcomes and did not adjust for multiple comparisons.

**Table 2** presents the observed values at each visit and the change within each group. For corneal nerve fiber density, the mean changed by −0.66 fibers/mm² in the PF HA group (95% confidence interval [CI], −5.11 to 3.79) and by 2.86 fibers/mm² in the BAK HA group (95% CI, −2.01 to 7.74). For corneal nerve fiber length, the corresponding changes were −0.09 mm/mm² (95% CI, −1.76 to 1.58) and 0.95 mm/mm² (95% CI, −1.07 to 2.97). For these two parameters, the point estimates of the within-group changes had opposite signs. None of the fourteen within-group changes had a confidence interval that excluded zero. For each parameter, the between-group difference in **Table 1**corresponds to the difference between the two within-group changes in **Table 2**.

## Discussion

In this substudy of a randomized, single-masked, six-month trial, six months of treatment with BAK-preserved hyaluronic acid was not associated with a detectable change in any of the seven corneal subbasal nerve parameters measured by IVCM, compared with the preservative-free formulation. Because the baseline parameters were comparable between the two groups, the absence of a between-group difference at six months cannot be attributed to an imbalance in corneal innervation at enrollment.

The participants analyzed here constitute a subgroup of the cohort in which the trial assessed clinical outcomes [48]. In that analysis, PF HA produced a greater reduction in the OSDI© score than BAK HA (adjusted change from baseline to the final visit, −14.36 versus −9.42; P = 0.001). Approximately 40% of participants treated with PF HA reached a normal OSDI© score at the final visit, whereas none of those treated with BAK HA did. Participants treated with PF HA instilled fewer drops per day (P < 0.001). Clinical signs improved in both groups, and we observed no significant between-group difference at the final visit. We obtained these symptomatic outcomes in the full completer cohort of 43 participants rather than the 32 analyzed here, and we did not perform an individual-level analysis linking symptoms to nerve morphology. Therefore, the relationship between the structural findings reported here and the symptomatic improvement remains undetermined.

Experimental evidence shows that BAK damages corneal nerves in a dose-dependent manner. Chen and colleagues applied 0.005%, 0.01%, or 0.02% BAK once daily to rabbit eyes for nine days and observed reductions in superficial intraepithelial nerve density of 58%, 67%, and 71% by day 3; involvement of the subbasal plexus by day 6; and a 52% reduction in stromal nerve fiber density at the highest concentration by day 9. Nerve density assessments were derived from cornea whole‐mount immunostaining, whereas in vivo confocal imaging assessed the structure of the central cornea on day 9 [38]. Sarkar and colleagues used Thy1-YFP reporter mice and observed reduced nerve fiber density after one week of treatment with 0.1% BAK, along with increases in markers of axonal injury and regeneration, indicating degeneration followed by sprouting rather than simple loss [39]. Komai and colleagues showed that a single instillation of 0.1% BAK on a debrided murine cornea reduces stromal nerve volume and corneal mechanosensation [40], and Ivakhnitskaia and colleagues showed that continuous exposure at clinically used concentrations impairs the activity of corneal cold-sensitive neurons in the rat, without recovery after washout [41].

Three differences between these experiments and the present trial affect interpretation. First, the concentrations used in animal studies were equal to or higher than the 0.005% in the formulation used here, and the highest exceeded it by up to a factor of twenty. Second, the animals received a fixed daily dose. In contrast, our participants instilled the drops pro re nata, with group means ranging from 2.67 to 4.22 instillations per day across follow-up visits in the full cohort [48]. Third, the animals were exposed to BAK in a saline vehicle or a hypotensive formulation, whereas our participants were exposed to BAK in a hyaluronate-containing artificial tear. Vereertbrugghen and colleagues reported that sodium hyaluronate attenuates BAK-associated epithelial and neuronal toxicity in mice and accelerates the recovery of corneal mechanical sensitivity [42]. The present study did not test that mechanism, so the vehicle’s protective contribution remains possible rather than demonstrated. Species differences in susceptibility further limit extrapolation from these models to patients.

The human evidence on preservatives and corneal nerves comes largely from patients receiving chronic topical therapy for glaucoma. Martone and colleagues examined 84 patients treated for at least 12 months. They reported fewer subbasal nerve trunks, more bead-like formations, and greater tortuosity in the groups receiving preserved medications than in the control group, with intermediate values in the preservative-free group [43]. Villani and colleagues examined 100 patients with medically controlled glaucoma and 50 controls. They reported increased total traced subbasal nerve length, increased tortuosity, and increased dendritic cell density in treated patients, with the magnitude of the changes related to the number of drugs and daily instillations [44]. Agnifili and colleagues reported reductions in corneal nerve fiber density, fiber length, and branch density in patients receiving multiple medications, together with increased dendritic cell density that correlated with the OSDI© score [45]. Using automated analysis, Zhu and colleagues reported that corneal nerve fiber density, branch density, fiber length, total branch density, fiber area, and fractal dimension declined and that fiber width increased as the number of medications and daily instillations rose [46]. Rossi and colleagues followed glaucoma patients for 36 months with masked confocal assessments and observed no measurable structural degradation with preservative-free monotherapy [52]. In all these studies, exposure to BAK was accompanied by an active hypotensive drug, and in many by several such drugs, so the preservative’s independent contribution cannot be separated from that of the active compound. Our design removes the active drug from the comparison.

Several explanations may account for the absence of a detectable difference, and the measurement itself is the first. Each parameter was derived from a single 400 by 400 micrometer frame per eye and per visit, sampling 0.16 mm² of the central subbasal nerve plexus. Vagenas and colleagues showed that five randomly selected, minimally overlapping central images are needed for a corneal nerve fiber length estimate to fall within 13% of the value obtained from 16 images 80% of the time, and that eight images are needed for branch density to fall within 30% of that value 95% of the time [49]. Analyzing expanding mosaic images, Allgeier and colleagues reported that corneal nerve fiber length varies considerably below a sampled area of about 1.5 mm² and stabilizes above it [53]. Applying a standardized selection protocol to six images per participant, Kalteniece and colleagues obtained intraclass correlation coefficients above 0.93 for fiber density, branch density, and fiber length [50]. No single number of fields applies to every protocol, but a sampled area of 0.16 mm² lies below the values examined in these studies. Therefore, a single frame per eye limits the precision with which we could detect a between-group difference, and this is the principal methodological limitation of the present analysis.

Exposure and duration give further explanation. The concentration used here was 0.005%, and the patient determined the regimen rather than a fixed schedule. We did not compute the cumulative dose of preservative delivered to each participant, and a pro re nata regimen does not by itself guarantee a lower cumulative exposure than a fixed one. Six months may also be too short. Fernández Jiménez-Ortiz and colleagues compared BAK-preserved with BAK-free hypotensive drops over a mean of approximately 22 weeks and likewise found no difference in subbasal nerve branching [54]. In contrast, studies reporting nerve differences generally involved 12 months or more of treatment.

The analysis was also restricted to the central cornea, so changes confined to the inferior whorl or the peripheral plexus would not have been captured [55]. Finally, corneal nerve parameters measured by IVCM vary considerably between individuals, as the standard deviations in **Table 4** show, and this variation reduces the precision achievable in a subgroup of this size.

Therefore, estimate precision matters as much as statistical significance. For corneal nerve fiber density, the confidence interval ranged from a change of 2.32 fibers/mm² smaller in the BAK HA group to a change of 9.37 fibers/mm² larger in that group; the interval is not symmetric about zero, and the data are compatible with a considerably larger difference in one direction than in the other. The study was not designed to demonstrate equivalence or non-inferiority, and these results should be read as an absence of evidence for a preservative-related change in corneal innervation at this exposure rather than as evidence that no such change occurs. The point estimates for six of the seven parameters were also positive, showing a numerically greater increase in the BAK HA group. **Table 2** shows that, for fiber density and fiber length, this pattern reflects a numerical increase in the BAK HA group and a numerical decrease in the PF HA group. Since none of these estimates reached statistical significance and every confidence interval included zero, this pattern should not be interpreted as a treatment effect. A comparable numerical pattern was observed for non-invasive tear break-up time in the clinical analysis of the same trial [48].

The strengths of this analysis include the randomized allocation; the use of two formulations with the same sodium hyaluronate concentration and molecular weight, produced by the same manufacturer; the six-month duration; the masking of both the operator who selected the images and the observer who analyzed them; and the analysis of both eyes of each participant using mixed-effects models which account for the resulting correlation. We should state the limitations equally plainly. The substudy was not powered for corneal nerve parameters; the parent trial’s sample size was calculated based on the OSDI© score [48]. Although we analyzed 62 eyes, the randomized units were the 32 participants. Eleven completers were not imaged, all of whom were women. Although each decision was made at the baseline visit and could not have been influenced by treatment, the resulting subgroup is not a random sample of the completer cohort, and women are underrepresented. We restricted measurement to a single central frame per eye, as discussed above. We did not assess measurement reproducibility in this cohort. Assessing it would have required repeated acquisition and independent frame selection rather than re-analysis of the same frame, so the variability contributed by acquisition and frame selection is unknown. Corneal sensitivity was not measured, so the structural findings cannot be related to a functional measure. Dendritic cell density, which has been reported to increase reversibly after exposure to BAK alone in healthy volunteers [47], was not quantified. Complete masking of participants was not feasible because the packaging of the two products differs. Finally, the follow-up was limited to six months, whereas patients with DED often continue topical therapy for years.

In clinical practice, these outcomes should be considered alongside the clinical results of the same trial. The preservative-free formulation provided greater symptomatic relief and required fewer daily instillations [48], and expert recommendations advise avoiding preservative-containing lubricants in patients who require frequent or long-term dosing [56,57]. The present analysis does not contradict that guidance. It shows only that, over six months and at the concentration used in artificial tears, we did not detect a difference between the two formulations in central corneal subbasal nerve parameters, given our available precision. Longer trials with larger samples, multiple fields sampled per eye, imaging of the inferior whorl in addition to the central cornea, and concurrent measurement of corneal sensitivity and dendritic cell density would be required to determine whether corneal nerve alterations develop with continued exposure.

## Conclusions

In this secondary IVCM analysis of a randomized trial, six-month changes in central corneal subbasal nerve parameters did not differ significantly between patients using 0.15% sodium hyaluronate containing 0.005% BAK and those using the preservative-free formulation, both on a pro re nata basis. The absence of statistically significant differences does not establish equivalence or rule out preservative-related effects on corneal nerves. For patients who need frequent or long-term treatment, preservative-free artificial tears remain a reasonable choice based on evidence regarding symptoms and ocular surface outcomes, rather than on the nerve measurements reported here.

## Data Availability

The data sets used and analyzed during the current study are available from the corresponding author upon reasonable request.
